# Effects of Supplemental Antioxidative Substances on Micronutrient Retention and Antioxidative Capacity in Rapeseed Oil During Low-Temperature Ethanol Steam Deodorization

**DOI:** 10.3390/foods14223907

**Published:** 2025-11-15

**Authors:** Fangrong Liu, Chengming Wang, Aifeng Niu, Yu Wang, Guowei Ling, Shilin Liu, Yuhan Yi, Mingshuang Xia

**Affiliations:** 1 College of Food Science and Technology, Huazhong Agricultural University, Wuhan 430070, China; 2 Key Laboratory of Environment Correlative Dietology, Huazhong Agricultural University, Ministry of Education, Wuhan 430070, China

**Keywords:** ethanol steam deodorization, rapeseed oil, antioxidative substances, micronutrients, antioxidative capacity, synergistic and/or antagonistic effects

## Abstract

This study investigated the effects of four antioxidative substances, α-tocopherol (α-TP), phytosterols (PS), squalene (SQ), and propyl gallate (PG), on micronutrient retention and antioxidative capacity of rapeseed oil during ethanol steam deodorization (ESD, 140–220 °C). Results demonstrated that supplementation with these antioxidants increased the retention rates (percentage relative to initial content) of tocopherols, phenols, carotenoids, phytosterols, and squalene by up to 2.92%, 46.25%, 25.82%, 1.03%, and 60.15%, respectively. These improvements are attributed to the protective effects of the supplemental antioxidative substances against thermal and oxidative degradation. Moreover, α-TP and PG increased the antioxidative capacity of the oil after ESD at 180 °C for 60 min by 10.37% and 5.35%, respectively, which can be attributed to their increased concentrations and synergistic interactions with endogenous antioxidants. A model oil system of caprylic triglyceride supplemented with binary mixtures of antioxidative substances revealed synergistic behavior in blends of α-TP with PG or CE (β-carotene), and of CE with PG or PS, through complementary interactions, whereas antagonism occurred in blends of PS with PG, and of SQ at a high concentration (40.10 mg/100 g) with α-TP, PG or CE, due to unfavorable molecular interactions.

## 1. Introduction

Vegetable oils contain a range of micronutrients, such as tocopherols, polyphenols, phytosterols, squalene, and carotenoids, which are crucial for human health despite their concentrations typically being below 1% [1]. Tocopherols and polyphenols exhibit a variety of biological activities such as improving cardiovascular health, preventing cataract formation, and reducing the risk of neurodegenerative diseases and cancer [2,3]; phytosterols reduce cholesterol levels within the organism and demonstrate neuroprotective effects against neurodegenerative diseases [4]. Moreover, these micronutrients can act as endogenous antioxidative substances by leveraging their antioxidative activity to inhibit lipid oxidation. For instances, α-tocopherol (α-TP) can improve the oxidative stability of oil by inhibiting lipid peroxidation [5]; phytosterols (PS) can inhibit the processes of both autoxidation and photooxidation in oils [6]; phenolic compounds are capable of inhibiting oxidative degradation of oils via hydrogen atom transfer, single electron transfer, and transition metal chelation [7]; carotenoids such as β-carotene and xanthophyll can utilize the conjugated double bonds and the functional groups in their molecules to absorb light energy, thus effectively inhibiting the photo-oxidation of oils [8]; and squalene (SQ) can inhibit lipid peroxidation via the chain-breaking mechanism or quenching singlet oxygen [9]. Therefore, preserving more of these micronutrients during oil processing not only significantly improves the nutritional quality of the oil but also enhances its oxidative stability to prolong the shelf life.

Deodorization is a key step in the refining process of oil, aiming at removing undesirable flavor compounds (e.g., aldehydes, ketones, free fatty acids, etc.) and harmful substances (e.g., polycyclic aromatic hydrocarbons, etc.) [10,11,12]. Studies have shown that low-temperature ethanol steam deodorization (L-ESD, 180 °C for 80–100 min) presents a superior alternative to conventional high-temperature water steam deodorization (H-WSD, 250 °C for 60 min) for rapeseed oil processing. Specifically, L-ESD better preserves valuable micronutrients, including tocopherols, polyphenols, phytosterols, and carotenoids, while simultaneously reducing undesirable compounds such as hydroperoxides, free fatty acids, and polycyclic aromatic hydrocarbons (PAHs), while also reducing decreasing processing contaminants like 3-chloropropanol esters and glycidyl esters by approximately 50% [13,14]. Among them, the better preservation of micronutrients in oil during the L-ESD process was attributed to their lower thermal oxidative degradation and other chemical changes due to the lower deodorization temperature in comparison with the H-WSD process [13,15].

Nevertheless, the reduction of micronutrients during deodorization results not only from the entrainment effect of ethanol steam in distillation, but also from chemical mechanisms, including oxidative degradation. Recent studies have shown that rosemary extract, sage extract and thyme extract significantly reduced the loss of γ-tocopherol in soybean oil and hempseed oil during heating at 180 °C [16]; besides, rosemary extract significantly reduced the loss of tocopherols in rapeseed oil during storage at 65 °C [17]. These findings suggest that antioxidative substances can effectively slow down the oxidative degradation of micronutrients in oil during the heating process, thus the supplement of antioxidative substances in oil during the deodorization process may be a viable strategy to inhibit the oxidative degradation of micronutrients, thereby improving the nutritional value of deodorized oil. Natural antioxidative substances such as α-TP, PS and SQ can be obtained by extraction from deodorized distillates of vegetable oils, which have significant advantages such as high safety and greenness compared with synthetic antioxidative substances [18]. Re-adding these endogenously antioxidative substances to vegetable oils can not only inhibit the oxidative degradation of unsaturated fatty acids [19,20], but also directly increase the content of these physiologically active substances in vegetable oils, further enhancing the nutritional quality of oils. Propyl gallate (PG), due to its biocatalytic synthesis, is less toxic and more environmentally friendly than traditional synthetic phenolic antioxidative substances, such as butylated hydroxyanisole, 2,6-di-tert-butyl-p-cresol, and tert-butylhydroquinone [21,22]. Therefore, it is a potential solution to add antioxidative substances such as α-TP, PS, SQ, or PG before deodorization to inhibit micronutrient degradation and safeguard the product’s nutritional value. However, the current understanding of how antioxidative substances influence micronutrients during deodorization is severely limited. Meanwhile, existing studies on these endogenous antioxidative substances often employ vegetable oils with stripped micronutrients as reaction media, which fails to replicate the complex matrix of real oils [23,24], and making it difficult to extrapolate the findings and assess their true protective efficacy and synergistic effects in real oils.

To further improve the retention of micronutrients in oil and the antioxidative capacity of oil after ESD, this paper investigated the effects of α-TP, PS, SQ, and PG on the retention rates of micronutrients, such as tocopherols, polyphenols, carotenoids, phytosterols, and squalene, in rapeseed oil during ESD at 140–220 °C for 60 min. Additionally, the effects of these antioxidative substances on the antioxidative capacity of rapeseed oil during ESD at 180 °C for 60 min was evaluated using 2,2-diphenyl-1-picrylhydrazyl (DPPH) scavenging rates. Meanwhile, a model oil system of caprylic triglyceride supplemented with binary mixtures of antioxidative substances was utilized to investigate their synergistic and/or antagonistic effects via DPPH radical scavenging rate and the resulting effects on rapeseed oil’s antioxidative capacity. The findings will provide theoretical basis and technological guidance for improving micronutrient retention and antioxidative capacity of the oil during ESD.

## 2. Materials and Methods

### 2.1. Materials and Reagents

The low-erucic-acid expeller-pressed rapeseed oil used in this study was purchased from a processing company in Chengdu, Sichuan Province, China. Its erucic acid content was approximately 3%, and its complete fatty acid composition was as follows: oleic acid (61.34% ± 0.44%), linoleic acid (18.69% ± 0.18%), linolenic acid (8.46% ± 0.18%), palmitic acid (5.19% ± 0.55%), octadecanoic acid (1.95% ± 0.07%), arachidic acid (1.75% ± 0.31%), and erucic acid (2.63% ± 0.83%). Caprylic triglyceride (purity ≥ 99%) was purchased from Sport Research, Inc. (San Pedro, CA, USA); α-tocopherol (purity ≥ 96%), phytosterols (purity ≥ 95%), squalene (purity ≥ 99%), propyl gallate (purity ≥ 98%), gallic acid (purity ≥ 99%), 2,2-diphenyl-1-picrylhydrazyl (purity ≥ 96%), N,O-Bis (trimethylsilyl) trifluoroacetamide with trimethylchlorosilane (BSTEA:TMCS = 99:1), and chromatographic reagents such as methanol and n-hexane were purchased from Macklin Bio-Technology Co., Ltd. (Shanghai, China). Standards of cholesterol (purity ≥ 98%) and β-carotene (purity ≥ 97%) were purchased from Shanghai Yuan Ye Bio-Technology Co., Ltd. (Shanghai, China). Standards of tocopherols (α-, β-, γ-, and δ-, purity ≥ 98%) were obtained from ANPEL Laboratory Technologies Inc. (Shanghai, China). Analytical-grade methanol, anhydrous ethanol, potassium hydroxide, and anhydrous sodium carbonate were obtained from Sinopharm Chemical Reagent Co., Ltd. (Shanghai, China).

### 2.2. Preparation of Deodorized Rapeseed Oil Samples

α-Tocopherol (50 mg/100 g oil), phytosterols (50 mg/100 g oil), squalene (50 mg/100 g oil), and propyl gallate (5 mg/100 g oil) were individually added to rapeseed oil. The oil samples were purged with nitrogen for 15 min to remove oxygen and then deodorized with ethanol steam following the previous deodorization method [15]. PRO (100 g) was placed into a three-necked flask, which was then positioned in an electric heating jacket (Wuhan Keer Instrument Co., Ltd., Wuhan, China) and connected to a mechanical vacuum pump. First, the oil was degassed with the vacuum pump, then heated to the target temperature (140, 160, 180, 200, and 220 °C). Preboiling anhydrous ethanol steam was introduced into the bottom layer of the oil. The entire deodorization process lasted for 60 min, with the ethanol steam flow rate maintained at 0.12 mL/min and the vacuum was maintained at 5 to 10 mbar. The deodorized oil was then cooled to a temperature of 50 °C under vacuum conditions before being stored at −20 °C for further analysis. Rapeseed oil samples without any supplemented antioxidative substances were designated as the CK group, while those supplemented with α-tocopherol, phytosterols, squalene, and propyl gallate were labeled as the α-TP, PS, SQ, and PG groups, respectively.

### 2.3. Preparation of Model Oil Samples

Two antioxidative substances were selected from α-TP, PG, PS, SQ and β-carotene (CE) and dissolved in caprylic triglyceride to form model oils with binary mixtures of antioxidative substances, and the concentration of each antioxidative substance corresponded to its respective level of concentration in rapeseed oil supplemented with the antioxidative substances after ESD at 180 °C for 60 min. The specific experimental design is shown in Table 1. The model oil samples were subsequently stored in the dark at −20 °C for spare use.

### 2.4. Determination of Tocopherols by Ultra Performance Liquid Chromatography (UPLC)

The content of tocopherols was determined following previously reported methods [25]. Oil samples (1.00 g) were dissolved in 10 mL n-hexane, and then the samples were filtered through a nylon filter with a pore size of 0.22 μm. A 20 μL aliquot of the filtered samples was injected into the UPLC system (Thermo Ultimate 3000; Thermo Fisher Scientific Ltd., Waltham, MA, USA), equipped with an AcclaimTM C30 column (250 mm × 4.6 mm ×3 μm, Thermo scientific). A mixture of methanol and water (80:20, *v*/*v*) was used as the mobile phase under isocratic conditions, with a flow rate of 0.80 mL/min, and the detection was performed at a wavelength of 294 nm using an ultraviolet detector (UVD). The chromatogram of tocopherol is shown in Figure S1 (Supplementary Material). The various tocopherol contents were quantified based on the calibration curves constructed from a series of methanol solutions containing α-, γ-, and δ-tocopherols at concentrations of 1.0, 2.5, 5.0, 10.0, 25.0, 50.0, and 100.0 μg/mL. The results were expressed in mg/kg oil, and the standard curves for the three types of tocopherols are as follows: α-tocopherol: y = 0.0992x + 0.0159, R^2^ = 0.9997; γ-tocopherol: y = 0.2006x + 0.0414, R^2^ = 0.9997; δ-tocopherol: y = 0.1412x − 0.0195, R^2^ = 0.9999.

### 2.5. Determination of Total Phenols by Spectrophotometry

The total phenol content in the oil was determined by spectrophotometry at 765 nm (UV-1800, Shimadzu, Tokyo, Japan) using the Folin–Ciocalteu reagent according to the previously described method [26]. The results were expressed as milligrams of gallic acid equivalents per kilogram of oil (mg GAE/kg oil), based on the gallic acid calibration curve, with the following equation: y = 0.0129x + 0.0204, R^2^ = 0.9987.

### 2.6. Determination of Total Carotenoids by Spectrophotometry

The total carotenoid content in the oil was determined following a previously described method [27]. Oil samples (0.5 g) were dissolved in 5 mL of n-hexane and analyzed using spectrophotometry at 445 nm (UV-1800, Shimadzu, Tokyo, Japan). Results were expressed as milligrams of β-carotene equivalents per kilogram of oil (mg β-CE/kg oil), based on the β-carotene calibration curve, with the following equation: y = 0.2175x + 0.0244, R^2^ = 0.9995.

### 2.7. Determination of Phytosterols and Squalene by Gas Chromatography

The content of phytosterols and squalene in the oil was analyzed following a previously established method [28]. A volume of 200 μL of 0.1% cholesterol solution (used as an internal standard) and 2 mL of 0.5 mol/L potassium hydroxide–ethanol solution were added to 0.25 g of oil sample. The mixture was then heated in a water bath at 80 °C for 30 min, vortexing briefly every 10 min. After cooling to room temperature, 1 mL of n-hexane was added to extract the unsaponifiable lipid fraction, followed by the addition of 1.5 mL of water to separate the two phases. The mixture was vigorously shaken and centrifuged at 4000 rpm for 10 min using an Anke TDL-5-A centrifuge (Anke, Shanghai, China). The upper organic phase was carefully transferred to a new tube (repeated three times) and evaporated to dryness under nitrogen. The derivatization reagent BSTEA:TMCS (99:1) (150 μL) was added to the tube, vortexed and mixed for 10 s, and heated at 70 °C for 30 min. After cooling, the sample was filtered through a nylon filter with a pore diameter of 0.22 μm, and 1 μL of the sample was injected into a gas chromatograph (Agilent Technologies 6890N; Agilent Technologies Company, Santa Clara, CA, USA) equipped with an HP-5MS column (30 m × 0.25 mm × 0.25 μm, Agilent Technologies Company, Santa Clara, CA, USA). The initial column temperature was set at 200 °C and held for 3 min, then increased to 220 °C at a rate of 2 °C/min, followed by a further increase to 300 °C at a rate of 20 °C/min and held for 15 min. The injector and detector temperatures were maintained at 300 °C. Nitrogen served as the carrier gas at a flow rate of 1.0 mL/min, with a sample split ratio of 15:1. The chromatogram of tocopherol is shown in Figure S2 (Supplementary Material). Based on Equation (1), phytosterols and squalene were quantified using cholesterol as the internal standard substance, and the results were expressed as milligrams of phytosterols or squalene per kilogram of oil (mg/kg oil).

(1) $$S=\frac{A_{0}\times m_{i}}{A_{i}\times m_{T}}\times 1000$$

where *S* is the content of phytosterol or squalene in the sample in mg/kg, *A_0_* is the peak area of phytosterol or squalene in the sample, *m_i_* is the mass of internal standard cholesterol in the sample (m = 0.2 mg), *A_i_* is the peak area of the internal standard cholesterol added to the sample, and *m_T_* is the mass of the sample in g.

### 2.8. Determination of 2,2-Diphenyl-1-Picrylhydrazyl Scavenging Capacity

The 2,2-diphenyl-1-picrylhydrazyl (DPPH) scavenging capacity of oil sample was determined according to the method described in previous studies [24]. A 0.1 g of oil sample was diluted with 4 mL of anhydrous ethanol. Then, 100 μL of the sample solution was mixed with 100 μL of 0.1 mmol/L DPPH solution in a 96-well plate and incubated in the dark at room temperature for 30 min. The absorbance of the mixture was measured at 517 nm using a microplate reader (Multiskan SkyHigh; Thermo Fisher Scientific Ltd., Waltham, MA, USA). The experimental scavenging capacity (ESC) was calculated using Equation (2):

(2) $$ESC=\frac{A_{0}-\left(A_{1}-A_{2}\right)}{A_{0}}\times 100\%$$

where *A*_0_, *A*_1_, and *A*_2_ are the absorbance values of the mixture of 100 μL anhydrous ethanol with 100 μL DPPH solution, the mixture of 100 μL sample solution with 100 μL DPPH solution, and the mixture of 100 μL sample solution with 100 μL anhydrous ethanol, respectively.

The theoretical scavenging capacity (TSC) is defined as the sum of the individual scavenging capacity of each equimolar substance in the mixture and calculated using Equation (3):

(3) $$TSC=\left({ESC}_{A}+{ESC}_{B}\right)-\frac{{ESC}_{A}\times {ESC}_{B}}{100}$$

where *ESC_A_* and *ESC_B_* are the experimental value of the individual minor constituents in the mixture.

### 2.9. Calculation of the Synergistic Effect (SE) of Antioxidative Substance Mixtures

The synergistic effect (SE) of binary mixtures is defined as the ratio of the experimental value to the theoretical value. The SE of binary mixtures of antioxidative substances was calculated according to the method described in previous studies [24], using Equation (4):

(4) $$SE=\frac{{ESC}_{m}}{{TSC}_{m}}$$

Accordingly, the antioxidative interaction of two antioxidative substances can be classified as a synergistic effect if their SE ratio > 1, an additive effect if their SE ratio = 1, and an antagonistic effect if their SE ratio < 1. Synergistic, additive or antagonistic effects were assigned on the basis of significant differences (*p* < 0.05) between the theoretical synergistic values and the experimental scavenging values.

### 2.10. Statistical Analysis

All analyses were performed in triplicate, and results are expressed as mean values ± standard deviations. One-way analysis of variance (ANOVA) was used to evaluate differences among groups, and Duncan’s multiple range test was applied to compare mean values using SPSS software (version 25.0, SPSS Inc., Chicago, IL, USA). Differences were considered statistically significant at *p* < 0.05.

## 3. Results

### 3.1. Effect of Antioxidative Substances on the Retention of Micronutrients in Rapeseed Oils

#### 3.1.1. Tocopherols

As shown in Table 2, three tocopherol isomers, namely α-, γ- and δ-tocopherols, were detected in pre-deodorized rapeseed oil (PRO), with concentrations of 277.15 mg/kg, 349.32 mg/kg, and 15.39 mg/kg, respectively. The content of tocopherols in ethanol team deodorization (ESD) rapeseed oil was gradually reduced as the deodorization temperature was increased from 140 to 220 °C. Moreover, the total retention of tocopherols in rapeseed oil varied among groups (Table 3), ranging from 95.93% to 89.07% for CK, from 95.48% to 90.74% for α-tocopherol (α-TP), from 98.17% to 91.67% for propyl gallate (PG), from 96.93% to 91.50% for phytosterols (PS), and from 96.92% to 89.84% for squalene (SQ). Among these, PG, PS and SQ caused a slight increase in the retention of total tocopherols compared to the CK group. Although the retention in the CK group was already high, the supplemental antioxidative substances provided a further enhancement, with effects in the order of PG > PS > SQ, which was attributed to the fact that (1) the PG molecule contains three phenolic hydroxyl groups on its benzene ring, which has strong antioxidative capacity and can protect tocopherols from oxidation [29], and (2) the PS could form a hydrogen bond with the tocopherols and reduce their distillation rate. In addition, PG, PS, and SQ can competitively scavenge lipid free radicals or singlet oxygen to reduce their attack on tocopherols during the ESD process. Notably, supplementation with α-TP had little effect on the retention of total tocopherols in oil. A detailed analysis of the isomers revealed that while the retention rate of α-tocopherol in the α-TP group was high (97.06–92.33%) after ESD at 140 to 220 °C, the retention of γ-tocopherol (91.99–87.72%) was lower than that in the CK group (96.03–88.78%). It is hypothesized that the addition of a high concentration of α-TP may have altered the interconversion or competitive degradation dynamics among tocopherol homologs, potentially promoting the oxidation of γ-tocopherol [30,31,32]. Consequently, the significant gain in α-tocopherol was largely offset by the greater loss of γ-tocopherol, resulting in the minimal observed change in the total tocopherol retention rate.

#### 3.1.2. Phenols

Phenolic compounds in rapeseed oil, such as phenolic acids and flavonoids [33], can capture the reactive groups in lipids to avoid the generation of oxidation products and stabilize oxygen radicals via intramolecular hydrogen bonding, which improves the oxidative stability of oils [34,35]. As shown in Table 2, the total phenol content in PRO was 86.27 mg GAE/kg, the total phenol content in ESD rapeseed oil in all groups decreased gradually with the increase in deodorization temperature from 140 to 220 °C (Table 3). The total phenol retention in rapeseed oil varied among groups, ranging from 66.63% to 13.21% for CK, from 68.26% to 10.79% for α-TP, from 69.69% to 16.82% for PG, from 73.26% to 18.98% for PS, and from 71.43% to 17.38% for SQ, indicating that PG, PS and SQ supplementation can increase the retention rate of total phenols in rapeseed oil during ESD. In the PG group, the retention rate of total phenols after ESD at 140–220 °C was 4.58–46.25% higher than that of the CK group, which was attributed to the fact that PG could protect the endogenous phenolic compounds from degradation and form intermolecular hydrogen bonds with these endogenous phenolic compounds [36], thus decreasing their volatilized loss during the oil deodorization process. Similarly, the PS and SQ groups showed 9.95–43.66% and 7.20–31.51% higher retention of total phenols than the CK group after ESD at 140–220 °C, respectively, which was attributed to the fact that they competed with phenolic compounds for scavenging of free radicals and singlet oxygen [6,9]; although SQ containing six 2-methyl-2-pentene units has a stronger antioxidative capacity than PS containing one double bond [6,37], PS enhanced the retention rate of phenolic compounds during ESD more than SQ. This is likely because PS can form hydrogen bonds with phenolic compounds [38,39], which reduces the volatility of phenolic compounds [40], resulting in less distilled phenols during the ESD process.

However, the retention rate changes of total phenols in rapeseed oil supplemented with α-TP (46.99–10.79%) were lower than that in the CK group (48.97–13.21%) with the temperature increased from 160 to 220 °C, while the retention rate of total phenols (68.26%) in the α-TP group via ESD at 140 °C was slightly higher than that of the CK group (66.63%), which might be attributed to the pro-oxidant effect of α-TP under elevated temperatures. During ESD between 160–220 °C, α-TP, after donating a hydrogen atom to scavenge a lipid radical, is converted into a tocopheroxyl radical, and this tocopheroxyl radical may itself promote the oxidation of other sensitive compounds, such as polyphenols [41]. Moreover, in the temperature range of 160 to 220 °C, the generation of these free radicals in the α-TP group increased with increasing temperature, which led to a greater loss of phenolic compounds, although the hydrogen bonds formed between the supplied α-TP and phenolic compounds could reduce the volatility of the phenolic compounds.

#### 3.1.3. Carotenoids

Carotenoids, characterized by a long carbon chain structure consisting of multi-conjugated double bonds, are effective energy-transfer quenchers of singlet oxygen [6], and their degradation products generated by isomerization and other reactions at high temperatures can also able to participate in free radical scavenging reactions [42]. As shown in Table 2, the total carotenoid content in PRO was 48.06 mg β-GE/kg, and the retention rate of total carotenoids in rapeseed oil varied among groups with the deodorization temperature increasing from 140 to 220 °C, ranging from 87.16% to 8.52% for CK, from 93.66% to 9.02% for α-TP, from 91.01% to 8.89% for PG, from 90.86% to 8.81% for PS, and from 91.71% to 8.76% for SQ (Table 3). The retention rates of total carotenoids in α-TP, PG, PS, and SQ groups were higher than those in CK group by 7.46–25.82%, 4.42–15.05%, 2.96–6.55%, and 5.22–12.31%, respectively, indicating that all four antioxidative substances can improve the retention rate of carotenoids, with their effectiveness in the order of α-TP > PG ≈ SQ > PS. Although PG exhibits stronger antioxidative capacity than α-TP, the protective effect of α-TP on carotenoids was more pronounced than that of PG, likely due to its significantly higher addition of α-TP than that of PG. Furthermore, SQ and PG showed similar effects on the retention rate of carotenoids in rapeseed oil, which can be attributed to the strong singlet oxygen quenching ability of the double bonds in SQ [9]. Additionally, the lowest increment in carotenoid retention rate in the PS group was attributed to the weaker antioxidative capacity of PS, which is primarily because their alcoholic hydroxyl group is a less active hydrogen donor in the prevailing hydrogen atom transfer (HAT) mechanism compared to phenolic antioxidative substances [43].

#### 3.1.4. Phytosterols

As shown in Table 2, PRO contained three phytosterols, namely brassicasterol, campesterol, and β-sitosterol, with the concentrations of 947.62 mg/kg, 2697.12 mg/kg, and 4300.65 mg/kg, respectively. The loss of phytosterols in rapeseed oil after ESD at 140–220 °C for 60 min was relatively low, and the retention of total phytosterols varied among groups, ranging from 98.96% to 94.34% for CK, from 98.52% to 94.66% for α-TP, from 99.29% to 94.85% for PG, from 99.12% to 94.91% for PS, and from 98.63% to 94.10% for SQ (Table 4), indicating a minimal impact of deodorization temperature and antioxidative substances such as α-TP, PS, SQ and PG (≤1%, relative to the CK group) on the retention rate of phytosterols during the ESD process. This is likely due to the relatively superior thermal and oxidative stability of phytosterols [44,45], and the negligible influence of hydrogen bonds between the hydroxyl group (-OH) of the phytosterols and the phenolic hydroxyl groups (-OH) of the added antioxidative substances (α-TP and PG) on phytosterol retention in the oil, given their lower concentrations compared to that of phytosterols in the oil.

#### 3.1.5. Squalene

As presented in Table 2, the squalene content in PRO was 32.67 mg/kg. The retention rate of squalene in CK, α-TP, PG, PS, and SQ groups decreased from 74.62% to 43.73%, 80.90% to 50.98%, 78.65% to 53.15%, 75.35% to 49.83%, and 91.09% to 69.49%, respectively, as the deodorization temperature was increased from 140 to 220 °C (Table 4). The retention rates of squalene in α-TP, PG, PS, and SQ groups were higher than those in the CK group by 0.69–16.57%, 5.41–21.54%, 0.99–13.94%, and 22.07–60.15%, respectively, suggesting that the supplementation of α-TP, PG, PS and SQ is capable of improving the retention rate of squalene in rapeseed oil, with efficacy in the order of SQ > PG > α-TP ≈ PS. Among them, the high squalene content resulting from SQ supplementation significantly improved the retention rate of squalene in rapeseed oil, even in the presence of a certain amount of oxygen. In addition, due to the presence of multiple unsaturated double bonds, squalene is highly susceptible to thermal and photochemical degradation accompanied by oxidation, so antioxidative substances such as α-TP, PS and PG can protect squalene from oxidative degradation and thus improve its retention during ESD. Given the molecular structure of PS, which are not efficient hydrogen donors, their protection of squalene likely involves indirect mechanisms. The bulky hydrocarbon structure of PS could potentially modify the oil matrix to create a less oxidative microenvironment or weakly intercept propagation radicals, which would collectively reduce the oxidative degradation of the more susceptible squalene.

In summary, α-TP supplementation in rapeseed was more effective in improving the retention rate of endogenous carotenoids and squalene in rapeseed oil during ESD; PG supplementation in rapeseed oil was more effective in the retention of total phenols, squalene and carotenoids; PS supplementation in rapeseed oil was more effective in the retention of total phenols and squalene; and SQ supplementation in rapeseed oil was more effective in the retention of squalene, carotenoids and total phenols. Therefore, the addition of α-TP, PS, SQ and PG can improve the retention rate of micronutrients in rapeseed oil during actual production.

### 3.2. Antioxidative Capacity of Rapeseed Oil Supplemented with Different Antioxidative Substances After ESD

The DPPH radical scavenging activity is a key indicator that reflects the content and overall antioxidative capacity of antioxidative substances in oils, and it is closely linked to the oxidative stability and storage life of the oils. While previous research has confirmed the efficacy of ESD at 180 °C, its potential for enhancing the antioxidative properties of rapeseed oil has not been fully elucidated. Therefore, this study further determined the DPPH scavenging capacity of rapeseed oil supplemented with various antioxidative substances after ESD at 180 °C for 60 min, aiming to elucidate whether these supplement antioxidative substances could synergistically enhance the oil’s antioxidant activity during ESD, thereby achieving a breakthrough improvement in the oxidative stability of the final product.

**Figure 1:**
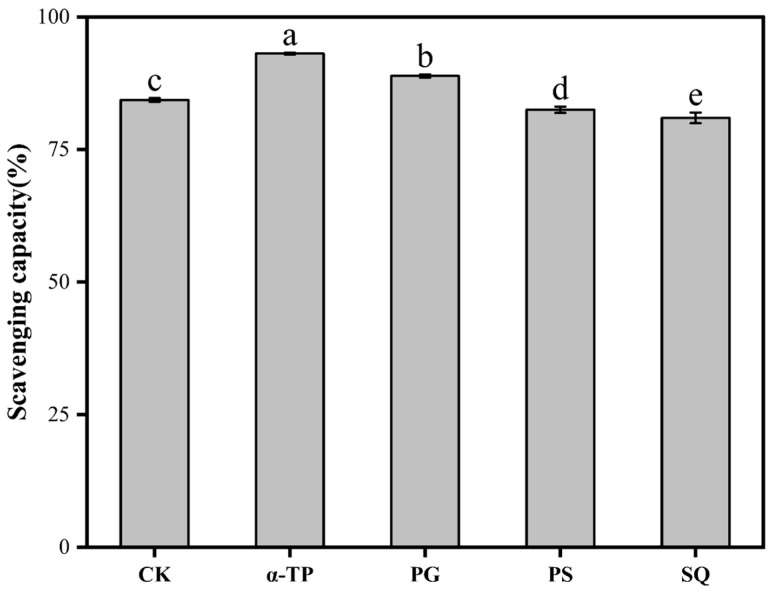
DPPH scavenging capacity of rapeseed oil and rapeseed oil supplemented with different antioxidative substances. Abbreviations: α-TP, α-tocopherol; PG, propyl gallate; PS, phytosterols; SQ, squalene. a–e mean significance analysis of scavenging capacity of rapeseed oil supplemented with different antioxidative substances after ESD at 180 °C for 60 min, and different letters indicate significant differences (*p* < 0.05).

As shown in Figure 1, the DPPH scavenging rates of rapeseed oil supplemented with α-TP (50 mg/100 g), PG (5 mg/100 g), PS (50 mg/100 g) and SQ (50 mg/100 g) were ranked as follows: α-TP (93.09%) > PG (88.85%) > CK (84.34%) > PS (82.48%) > SQ (80.94%). Here, the amount of PG supplemented was within the maximum allowable content (10 mg/100 g) for edible oils and fats as per Chinese standard GB 2760-2024 [46], a limit which is established based on toxicological assessments to prevent potential health risks [47]. This level is also well below the Acceptable Daily Intake (ADI) of 0–1.4 mg/kg body weight set by the Joint FAO/WHO Expert Committee on Food Additives (JECFA) [48], confirming that the estimated dietary exposure poses no foreseeable health risk. The DPPH scavenging rate increased by 10.37% in the α-TP-supplemented oil and by 5.35% in the PG-supplemented oil compared to the CK, which correlated with improved retention of their respective antioxidants: α-tocopherol retention was higher in the α-TP group (95.56%) than in the CK (94.30%), and total phenol retention was greater in the PG group (36.29%) than in the CK (24.81%). It is worth noting that the supplement of PS and SQ actually reduced the DPPH scavenging capacity of rapeseed oil by 2.21% and 4.03% compared to the CK group, respectively, despite the higher retention rates of total phenols and other endogenous nutrients in the PS and SQ groups (3.1.). Critically, it is well-established in the literature that PS and SQ exhibit negligible direct radical scavenging activity in the DPPH assay [9,49]. Therefore, their supplementation does not directly contribute to the DPPH scavenging capacity the observed decline suggests that PS and SQ may interfere with the activity of potent endogenous antioxidative substances (e.g., tocopherols and phenols) present in the rapeseed oil. These results indicate that the antioxidative interaction of PS or SQ with potent endogenous antioxidants in the complex matrix of rapeseed oil is not simply synergistic but potentially antagonistic. PS or SQ may disrupt the radical chain-breaking activity of these endogenous antioxidative substances, thereby suppressing their efficacy and ultimately leading to a decline in the overall antioxidant capacity of rapeseed oil.

### 3.3. Synergistic and/or Antagonistic Effects Between Antioxidative Substances in Model Oils

As mentioned above, there may be an antagonistic effect of PS or SQ with endogenous antioxidative substances in rapeseed oil, and the enhancement of antioxidative capacity in the α-TP and PG groups may also be due to the synergistic effect of supplemented α-TP or PG with endogenous antioxidative substances. To elucidate the effects of synergistic and/or antagonistic effects between supplemented antioxidative substances and endogenous antioxidative substances in rapeseed oil on the antioxidative capacity of rapeseed oil, the antioxidative interaction types of some binary mixtures of antioxidative substances among α-TP (representing α-tocopherol), PG (representing phenols), PS (representing β-sitosterol), SQ (representing squalene), and CE (β-carotene, representing carotenoids) were investigated by using caprylic triglyceride as the model oil. The synergistic and/or antagonistic effects of antioxidative substances were characterized via the synergistic effect (SE) of binary mixtures of antioxidative substances. The type of interaction between antioxidative substances (synergism or antagonism) depends on their concentration and ratio. The binary mixtures in this study were designed strictly based on the measured concentrations of various micronutrients in corresponding rapeseed oil supplement with antioxidative substances after ESD at 180 °C for 60 min, and the interaction results of this model can reflect to some extent the synergistic and/or antagonistic effects between antioxidative substances in rapeseed oil supplement with antioxidative substances.

**Figure 2:**
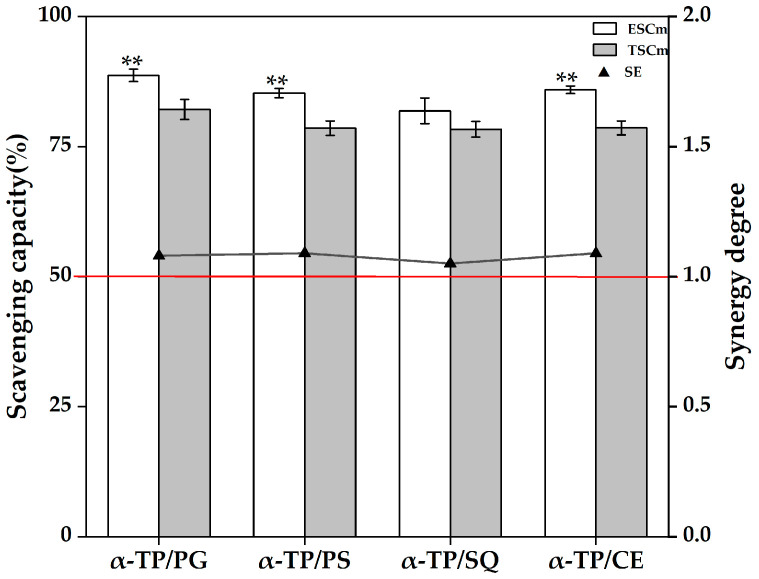
DPPH scavenging capacity and synergistic effects of binary mixtures of antioxidative substances in the α-TP model group. Abbreviations: α-TP, α-tocopherol; PG, propyl gallate; PS, phytosterols; SQ, squalene; ESCm, the experimental DPPH scavenging capacity of the binary mixtures of antioxidative substances; TSCm, the theoretical DPPH scavenging capacity of the binary mixtures of antioxidative substances; SE, the synergistic effects of binary mixtures of antioxidative substances. The abscissa represents the constituent components of the binary mixtures of antioxidative substances. Different asterisks indicate statistically significant differences between ESCm and TSCm (** *p* < 0.01).

As shown in Figure 2, the SE values for the binary mixtures of α-TP with PG (α-TP/PG), PS (α-TP/PS), or CE (α-TP/CE) in the α-TP model group were all greater than 1 (*p* < 0.05), indicating that the combination of α-TP (77.96 mg/100 g) with PG (2.00 mg/100 g), PS (414.72 mg/100 g), or CE (2.45 mg/100 g) exhibits a synergistic effect in model oil. The synergism between α-TP and PG suggests that PG may regenerate α-tocopherol by reducing its radical species, a mechanism demonstrated at oil–water interfaces in emulsion studies [50] and potentially occurring in the microenvironments of model oil system in this study. The synergism of α-TP with PS or CE, as reported in prior studies [24,51], suggests the presence of interactions that extend the antioxidant activity of α-TP. These interactions may involve mechanisms such as the stabilization of the tocopheroxyl radical or other cooperative pathways, although their precise nature has not been fully elucidated. Additionally, the combination of α-TP (77.96 mg/100 g) with SQ (1.83 mg/100 g) displays an additive effect (SE ≈ 1, *p* > 0.05).

**Figure 3:**
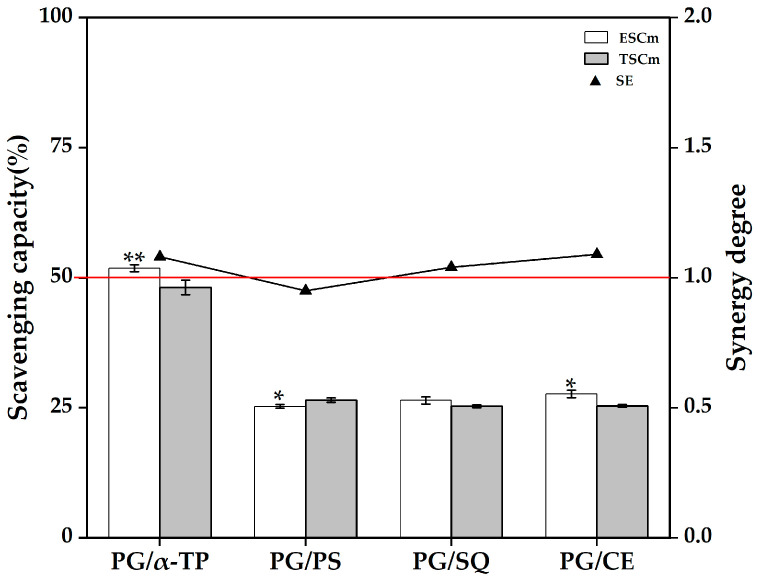
DPPH scavenging capacity and synergistic effects of binary mixtures of antioxidative substances in the PG model group. Abbreviations: α-TP, α-tocopherol; PG, propyl gallate; PS, phytosterols; SQ, squalene; ESCm, the experimental DPPH scavenging capacity of the binary mixtures of antioxidative substances; TSCm, the theoretical DPPH scavenging capacity of the binary mixtures of antioxidative substances; SE, the synergistic effects of binary mixtures of antioxidative substances. The abscissa represents the constituent components of the binary mixtures of antioxidative substances. Different asterisks indicate statistically significant differences between ESCm and TSCm (* *p* < 0.05, ** *p* < 0.01).

As shown in Figure 3, the SE values of the binary mixtures of PG with α-TP (PG/α-TP) or CE (PG/CE) in the PG model group were greater than 1 (*p* < 0.05), suggesting that PG synergizes with either α-TP (26.91 mg/100 g) or CE (2.41 mg/100 g) as the PG concentration is increased to 4.34 mg/100 g. Conversely, the ESCm of the binary blend of PG with PS (PG/PS) was lower than the TSCm (SE < 1, *p* < 0.05), indicating that the combination of PG (4.34 mg/100 g) with PS (422.57 mg/100 g) in the PG model group exhibits an antagonistic effect. This antagonism can be attributed to the pronounced disparity in molar concentration between PS (422.57 mg/100 g) and PG (4.34 mg/100 g). A plausible hypothesis is that at this high ratio, molecular interactions (e.g., hydrogen bonding) between PS and PG could reduce the electron density and, consequently, the hydrogen-donating capacity of PG’s phenolic hydroxyl groups. This proposed decrease in reactivity would account for the observed impediment in DPPH radical scavenging. The verification of this specific interaction, however, would require further dedicated spectroscopic studies. Additionally, the combination of PG (4.34 mg/100 g) with SQ (1.91 mg/100 g) exhibits an additive antioxidative effect (SE ≈ 1, *p* > 0.05).

**Figure 4:**
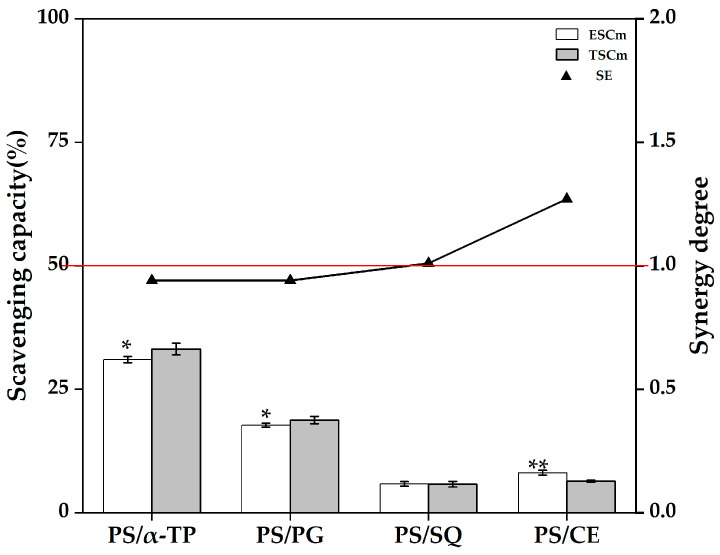
DPPH scavenging capacity and synergistic effects of binary mixtures of antioxidative substances in the PS model group. Abbreviations: α-TP, α-tocopherol; PG, propyl gallate; PS, phytosterols; SQ, squalene; ESCm, the experimental DPPH scavenging capacity of the binary mixtures of antioxidative substances; TSCm, the theoretical DPPH scavenging capacity of the binary mixtures of antioxidative substances; SE, the synergistic effects of binary mixtures of antioxidative substances. The abscissa represents the constituent components of the binary mixtures of antioxidative substances. Different asterisks indicate statistically significant differences between ESCm and TSCm (* *p* < 0.05, ** *p* < 0.01).

The binary blend of PS (455.54 mg/100 g) with α-TP (26.46 mg/100 g, PS/α-TP) in the PS model group exhibited an antagonistic effect (SE < 1, Figure 4), whereas α-TP/PS displayed a synergistic effect (SE > 1, Figure 2). This clear reversal demonstrates that the interaction between α-TP and PS is highly concentration-dependent. It is proposed that this switch can be explained by the thermodynamic feasibility of PS regenerating α-TP from its tocopheroxyl radical, a mechanism underpinned by their differing redox potentials [8,52]. Under conditions of high PS (455.54 mg/100 g) and low α-TP (26.46 mg/100 g) concentration (PS/α-TP), this regeneration is hypothesized to be outpaced by dominant inhibitory interactions (e.g., molecular crowding), leading to antagonism. Conversely, under conditions of relative low PS (414.72 mg/100 g) and high α-TP (77.96 mg/100 g) concentration (α-TP/PS), the regenerative interaction is posited to prevail, resulting in the observed synergy. Similar to the SE of PG/PS in the PG model group, the SE of the binary blend of PS with PG (PS/PG) in the PS model group was also less than 1 (*p* < 0.05), indicating that the combination of PS (455.54 mg/100 g) with PG (2.59 mg/100 g) exhibits an antagonistic effect, which can be explained by the same principle of unfavorable concentration ratios and molecular interactions. For the binary blend of PS (455.54 mg/100 g) with CE (2.14 mg/100 g, PS/CE) in the PS model group, the SE was greater than 1 (*p* < 0.05), indicating a synergistic effect of PS and CE in the PS group. This positive interaction could be due to complementary radical scavenging mechanisms or other beneficial interactions within the oil matrix. In addition, the binary blend of high PS concentration (455.54 mg/100 g) and low SQ concentration (1.74 mg/100 g) in the PS model group showed an additive effect (SE ≈ 1, *p* > 0.05).

**Figure 5:**
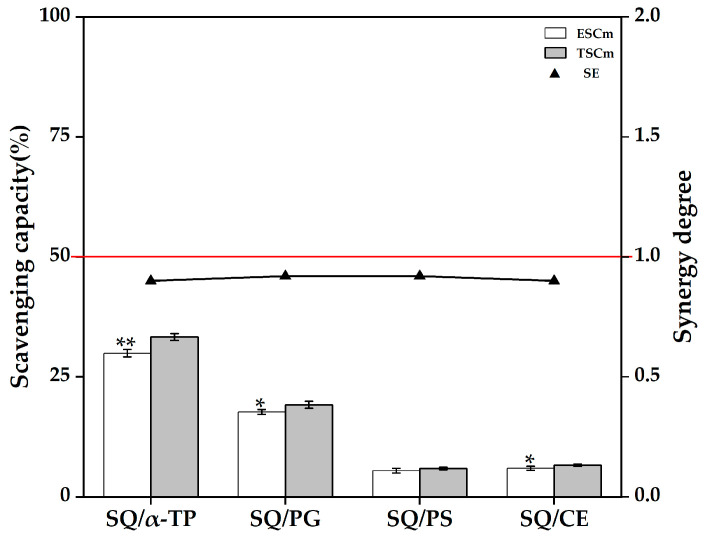
DPPH scavenging capacity and synergistic effects of binary mixtures of antioxidative substances in the SQ model group. Abbreviations: α-TP, α-tocopherol; PG, propyl gallate; PS, phytosterols; SQ, squalene; ESCm, the experimental DPPH scavenging capacity of the binary mixtures of antioxidative substances; TSCm, the theoretical DPPH scavenging capacity of the binary mixtures of antioxidative substances; SE, the synergistic effects of binary mixtures of antioxidative substances. The abscissa represents the constituent components of the binary mixtures of antioxidative substances. Different asterisks indicate statistically significant differences between ESCm and TSCm (* *p* < 0.05, ** *p* < 0.01).

As shown in Figure 5, the SE values of the binary mixtures of SQ with α-TP (SQ/α-TP), PG (SQ/PG), and CE (SQ/CE) in the SQ model group were all less than 1 (*p* < 0.05), indicating that a high concentration of SQ (40.10 mg/100 g) is antagonistic to a low concentration of α-TP (26.48 mg/100 g), PG (2.42 mg/100 g) or CE (2.37 mg/100 g). In contrast, α-TP/SQ (Figure 2) and PG/SQ (Figure 3) exhibited additive effects, these results demonstrated a concentration-dependent behavior in the antioxidative interaction patterns of SQ with α-TP or PG. The antagonism observed at high SQ concentrations could be due to its non-polar structure potentially interfering with the diffusion or collision efficiency of the smaller antioxidant molecules with free radicals. Similarly, the antagonism of high concentrations of SQ on CE may be related to the additive reaction between high concentrations of SQ and CE cation radicals. In addition, the high concentration of SQ (40.10 mg/100 g) with PS (421.56 mg/100 g, SQ/PS) exhibits an additive effect (SE ≈ 1, *p* > 0.05).

In summary, the changes in the DPPH scavenging capacity of the supplemented rapeseed oils can be rationalized by the specific interaction patterns observed in the model oil study. The decreased scavenging rate in the PS-supplemented oil is consistent with the antagonistic effect observed between PS and potent phenolic antioxidants in the model system. This antagonism, which likely involves molecular interactions that reduce the efficacy of phenolics, appears to occur during processing, as evidenced by the significant increase (20.65%) in the retention of total phenols compared to the CK group after ESD at 180 °C. This suggests that while PS may help retain phenolic compounds, it may simultaneously suppress their radical-scavenging activity. Any potential weak synergistic interaction between PS and endogenous carotenoids was insufficient to offset this overall decline in antioxidative capacity. Similarly, the reduced scavenging rate in the SQ-supplemented oil aligns with the antagonistic effects observed between high concentrations of SQ and low concentrations of key endogenous antioxidants (α-tocopherol, phenolics, and carotenoids) in the model oil, potentially due to physical interference with their reactivity. Conversely, the enhanced antioxidative capacity of rapeseed oil supplemented with α-TP or PG can be attributed to their respective synergistic effects with endogenous antioxidants, as confirmed in the model oil: α-TP with phenolic compounds, carotenoids, and β-sitosterol, and PG with α-tocopherol and carotenoids.

## 4. Conclusions

The supplementation of PG (5 mg/100 g) demonstrated the most significant improvement in the micronutrient retention rate of rapeseed oil during ESD at 140–220 °C for 60 min, with the incremental retention of tocopherols, total phenols, carotenoids, and squalene ranging from 2.29–2.92%, 4.58–46.25%, 4.28–15.05%, and 5.41–21.54%, respectively. This was followed by supplementation with SQ (50 mg/100 g), with the incremental retention of total phenols, carotenoids, and squalene ranging from 7.20–31.51%, 2.85–12.31%, and 22.07–60.15%, respectively. Supplementation with α-TP (50 mg/100 g) significantly increased the retention of carotenoids and squalene, while supplementation with PS (50 mg/100 g) significantly increased the retention of total phenols and squalene. Furthermore, α-TP and PG supplementation enhanced the antioxidative capacity of rapeseed oil by 10.37% and 5.35%, respectively, compared to the CK group during ESD at 180 °C for 60 min. Regarding the effects of synergistic and/or antagonistic effects on the antioxidative capacity in the DPPH scavenging reaction, α-TP exhibited synergistic effects with PG and CE; PG showed synergism with CE; and PS demonstrated synergism with CE. However, antagonistic effects were observed between PS and PG, and higher concentrations of SQ displayed antagonistic effects when combined with α-TP, PG, or CE. These synergistic and/or antagonistic effects between supplemented antioxidative substances and endogenous micronutrients in oil contribute to the antioxidative capacity of oil supplemented with antioxidative substances, besides the antioxidative capacity of supplementing antioxidative substances and endogenous micronutrients in oil during ESD. This study provides theoretical support for the selection of suitable antioxidative substances with a certain concentration to enhance the nutritional quality and antioxidative capacity of oil during ESD in actual production.

## Figures and Tables

**Table 1:** Contents of binary mixtures of antioxidative substances in model oil (mg/100 g).

Primary Substances	Paired Substances	α-TP	PG	PS	SQ	CE
α-TP	PG	77.96	2.00	-	-	-
α-TP	PS	77.96	-	414.72	-	-
α-TP	SQ	77.96	-	-	1.83	-
α-TP	CE	77.96	-	-	-	2.45
PG	α-TP	26.91	4.34	-	-	-
PG	PS	-	4.34	422.57	-	-
PG	SQ	-	4.34	-	1.91	-
PG	CE	-	4.34	-	-	2.41
PS	α-TP	26.46	-	455.54	-	-
PS	PG	-	2.59	455.54	-	-
PS	SQ	-	-	455.54	1.74	-
PS	CE	-	-	455.54	-	2.14
SQ	α-TP	26.48	-	-	40.10	-
SQ	PG	-	2.42	-	40.10	-
SQ	PS	-	-	421.56	40.10	-
SQ	CE	-	-	-	40.10	2.37

Abbreviations: α-TP, α-Tocopherol; PG, Propyl Gallate; PS, Phytosterols; SQ, Squalene; CE, Carotenoids. A dash (-) indicates that the substance was not present in the specific mixture.

**Table 2:** Micronutrient content in rapeseed oil and rapeseed oil supplemented with various antioxidative substances before ESD.

Components	PRO (CK)	α-T-PRO	PS-PRO	SQ-PRO	PG-PRO
α-Tocopherol (mg/kg)	277.15 ± 5.58 ^b^	815.82 ± 17.19 ^a^	278.63 ± 9.07 ^b^	278.91 ± 7.95 ^b^	277.02 ± 5.10 ^b^
γ-Tocopherol (mg/kg)	349.32 ± 6.19 ^b^	343.61 ± 7.86 ^a^	346.94 ± 5.49 ^b^	345.63 ± 5.80 ^b^	347.07 ± 8.05 ^b^
δ-Tocopherol (mg/kg)	15.39 ± 0.29 ^b^	15.28 ± 0.25 ^a^	15.35 ± 0.14 ^b^	15.44 ± 0.32 ^b^	15.48 ± 0.42 ^b^
Total tocopherols (mg/kg)	642.22 ± 5.67 ^b^	1174.71 ± 23.56 ^a^	641.27 ± 9.84 ^b^	640.34 ± 7.14 ^b^	639.92 ± 7.55 ^b^
Total phenols (mg GAE/kg)	86.27 ± 0.68 ^b^	86.08 ± 1.98 ^b^	86.61 ± 1.82 ^b^	85.41 ± 2.93 ^b^	119.66 ± 6.20 ^a^
Total carotenoids (mg β-CE/kg)	48.06 ± 0.15 ^a^	48.22 ± 0.41 ^a^	47.63 ± 0.60 ^a^	48.25 ± 0.34 ^a^	47.96 ± 0.92 ^a^
Brassicasterol (mg/kg)	947.62 ± 7.76 ^a^	937.87 ± 36.82 ^a^	965.42 ± 35.44 ^a^	952.24 ± 3.10 ^a^	947.62 ± 28.14 ^a^
Campesterol (mg/kg)	2697.12 ± 17.16 ^a^	2682.76 ± 34.13 ^a^	2743.54 ± 78.80 ^a^	2706.55 ± 43.23 ^a^	2703.05 ± 43.02 ^a^
β-sitosterol (mg/kg)	4300.65 ± 46.42 ^b^	4291.63 ± 12.15 ^b^	4814.89 ± 154.35 ^a^	4332.77 ± 96.39 ^b^	4304.25 ± 17.53 ^b^
Total phytosterols (mg/kg)	7945.41 ± 58.51 ^b^	7912.26 ± 17.08 ^b^	8382.33 ± 33.00 ^a^	7991.56 ± 75.53 ^b^	7954.92 ± 86.48 ^b^
Squalene (mg/kg)	32.67 ± 2.88 ^b^	32.56 ± 1.92 ^b^	32.51 ± 1.36 ^b^	529.70 ± 53.95 ^a^	32.55 ± 0.98 ^b^

Mean values ± standard deviation of determinations for triplicate samples. Abbreviations: PRO, pre-deodorized rapeseed oil; α-TP-PRO, pre-deodorized rapeseed oil supplemented with α-tocopherol; PS-PRO, pre-deodorized rapeseed oil supplemented with phytosterols; SQ-PRO, pre-deodorized rapeseed oil supplemented with squalene; PG-PRO pre-deodorized rapeseed oil supplemented with propyl gallate. ^a,b^ mean significance analysis of the content of same micronutrient in rapeseed oil supplemented with different antioxidative substances before ESD (*p* < 0.05).

**Table 3:** Retention rates of tocopherols, total phenols and Total carotenoids (%) in rapeseed oil and rapeseed oil supplemented with various antioxidative substances after ESD at different temperatures.

Temperature		Tocopherols			Total Tocopherols	Total Phenols	Total Carotenoids
(°C)		α-Tocopherol	γ-Tocopherol	δ-Tocopherol			
140	CK	96.18	96.03	91.36	95.93	66.63	87.16
	α-TP	97.06	91.99	92.47	95.48	68.26	93.66
	PG	98.32	96.76	94.71	98.17	69.69	91.01
	PS	98.06	95.59	92.97	96.93	73.26	90.86
	SQ	97.59	97.13	93.05	96.92	71.43	91.71
160	CK	95.07	93.32	88.11	93.87	48.97	73.26
	α-TP	96.23	90.31	89.27	94.28	46.99	82.95
	PG	97.08	95.16	90.76	96.01	53.63	80.81
	PS	96.34	94.37	90.76	95.60	63.20	78.05
	SQ	96.16	95.30	89.75	95.77	54.13	79.44
180	CK	94.30	91.18	87.26	92.64	24.81	43.72
	α-TP	95.56	89.94	87.43	93.99	23.20	50.87
	PG	97.14	93.13	88.82	95.21	36.29	50.30
	PS	95.76	92.14	88.87	93.75	29.94	45.02
	SQ	95.37	93.99	88.51	94.24	28.39	49.10
200	CK	92.51	89.74	86.87	90.91	17.49	14.01
	α-TP	93.98	88.71	88.15	92.78	16.29	17.62
	PG	95.29	91.74	88.88	93.16	24.56	15.29
	PS	95.27	90.29	88.22	91.94	23.66	14.75
	SQ	93.24	90.43	86.38	91.67	22.31	15.34
220	CK	90.50	88.78	86.22	89.07	13.21	8.52
	α-TP	92.33	87.72	86.32	90.74	10.79	9.02
	PG	92.96	90.86	86.55	91.99	16.82	8.89
	PS	92.99	90.01	84.44	91.50	18.98	8.81
	SQ	91.65	88.82	86.12	89.84	17.38	8.76

Abbreviations: α-TP, α-Tocopherol; PG, Propyl Gallate; PS, Phytosterols; SQ, Squalene. Retention rate of α-tocopherol in the α-TP group refers to the percentage of total α-tocopherol (endogenous + supplemented) remaining after processing, calculated as Final Concentration/Initial Total Concentration × 100%.

**Table 4:** Retention rates of phytosterol and squalene (%) in rapeseed oil and rapeseed oil supplemented with various antioxidative substances after ESD at different temperatures.

Temperature		Phytosterols			Total Phytosterols	Squalene
(°C)		Brassicasterol	Campesterol	β-Sitosterol		
140	CK	99.04	98.84	99.01	98.96	74.62
	α-TP	98.41	98.79	98.16	98.52	80.90
	PG	99.31	99.02	99.46	99.29	78.65
	PS	97.20	99.63	96.31	99.12	75.35
	SQ	99.05	99.34	98.08	98.63	91.09
160	CK	97.89	98.31	98.64	98.44	69.11
	α-TP	97.64	97.38	97.21	97.44	69.59
	PG	98.98	97.84	98.46	98.31	73.12
	PS	96.04	98.88	95.98	98.56	73.51
	SQ	97.57	97.71	97.26	97.45	88.52
180	CK	97.26	97.56	97.09	97.27	55.66
	α-TP	95.62	96.83	96.63	96.70	56.20
	PG	97.62	97.30	98.18	97.81	58.68
	PS	95.50	97.24	94.61	97.17	56.33
	SQ	96.64	97.33	97.30	97.23	75.70
200	CK	94.68	96.80	95.47	95.83	48.32
	α-TP	93.98	95.50	96.24	95.83	50.36
	PG	95.11	96.24	97.19	96.62	54.38
	PS	94.20	96.50	94.08	96.48	51.36
	SQ	95.70	96.40	97.31	96.81	77.38
220	CK	92.32	94.23	94.85	94.34	43.73
	α-TP	91.81	94.70	95.05	94.66	50.98
	PG	94.62	95.02	94.80	94.85	53.15
	PS	91.07	94.09	93.36	94.91	49.83
	SQ	94.49	94.23	93.93	94.10	69.49

Abbreviations: α-TP, α-Tocopherol; PG, Propyl Gallate; PS, Phytosterols; SQ, Squalene.

## Data Availability

The original contributions presented in the study are included in the article/Supplementary Material, further inquiries can be directed to the corresponding author.

## Supplementary Materials

The following supporting information can be downloaded at: https://www.mdpi.com/article/10.3390/foods14223907/s1. Figure S1. Liquid chromatogram of tocopherols in rapeseed oil. Figure S2. GC chromatogram of phytosterols in rapeseed oil.

## Abbreviations

The following abbreviations are used in this manuscript:

L-ESD	low-temperature ethanol steam deodorization
H-WSD	high-temperature water steam deodorization
PRO	pre-deodorized rapeseed oil
α-TP	α-tocopherol
PG	propyl gallate
PS	phytosterols
SQ	squalene
CE	β-carotene
DPPH	2,2-diphenyl-1-picrylhydrazyl
ESC	experimental scavenging capacity
TSC	theoretical scavenging capacity
SE	synergistic effect
