# Supplementary material for: Effects of Supplemental Antioxidative Substances on Micronutrient Retention and Antioxidative Capacity in Rapeseed Oil During Low-Temperature Ethanol Steam Deodorization
Source: Foods. 2025 Nov 15;14(22):3907. doi: 10.3390/foods14223907 (PMC12651763; doi:10.3390/foods14223907)
Supplement: Supplementary file 1 [file foods-14-03907-s001.zip › foods-3977537-supplementary.pdf]

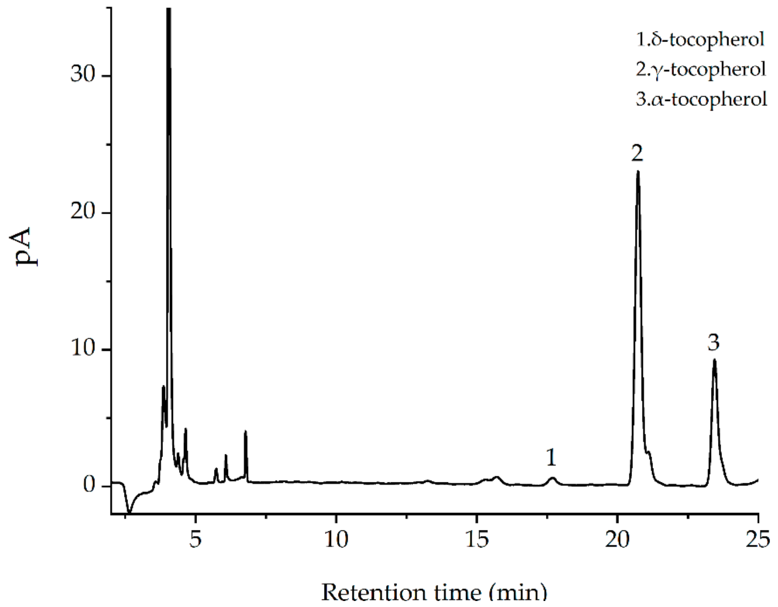

Figure S1. Liquid chromatogram of tocopherols in rapeseed oil

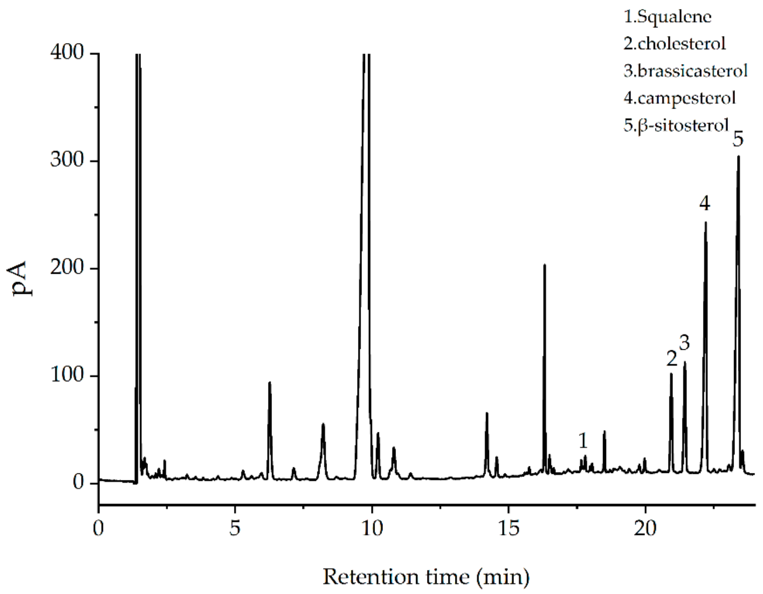

Figure S2. GC chromatogram of phytosterols in rapeseed oil
